# A Comprehensive Examination of Severely Ill ME/CFS Patients

**DOI:** 10.3390/healthcare9101290

**Published:** 2021-09-29

**Authors:** Chia-Jung Chang, Li-Yuan Hung, Andreas M. Kogelnik, David Kaufman, Raeka S. Aiyar, Angela M. Chu, Julie Wilhelmy, Peng Li, Linda Tannenbaum, Wenzhong Xiao, Ronald W. Davis

**Affiliations:** 1 ME/CFS Collaborative Research Center at Stanford, Stanford University School of Medicine, Palo Alto, CA 94305, USA; chiajung@stanford.edu (C.-J.C.); david@centerforcomplexdiseases.com (D.K.); raeka.aiyar@gmail.com (R.S.A.); amchu@stanford.edu (A.M.C.); wilhelmy@stanford.edu (J.W.); 2 Stanford Genome Technology Center, Stanford University School of Medicine, Palo Alto, CA 94304, USA; 3 Department of Biochemistry, Stanford University School of Medicine, Palo Alto, CA 94305, USA; 4 ME/CFS Collaborative Research Center at Harvard, Massachusetts General Hospital, Boston, MA 02114, USA; LHUNG1@mgh.harvard.edu (L.-Y.H.); pli6@mgh.harvard.edu (P.L.); 5 Basis Diagnostics, Newark, CA 94560, USA; andy@flexis.net; 6 Open Medicine Foundation, Agoura Hills, CA 91301, USA; ltannenbaum@omf.ngo; 7 Department of Genetics, Stanford University School of Medicine, Palo Alto, CA 94305, USA

**Keywords:** severe ME/CFS, quality of life, clinical symptoms, sleep, cognitive tests, laboratory tests, viral infection, antibody and antigen, long COVID, post-acute sequelae SARS-CoV-2 infection (PASC)

## Abstract

One in four myalgic encephalomyelitis/chronic fatigue syndrome (ME/CFS) patients are estimated to be severely affected by the disease, and these house-bound or bedbound patients are currently understudied. Here, we report a comprehensive examination of the symptoms and clinical laboratory tests of a cohort of severely ill patients and healthy controls. The greatly reduced quality of life of the patients was negatively correlated with clinical depression. The most troublesome symptoms included fatigue (85%), pain (65%), cognitive impairment (50%), orthostatic intolerance (45%), sleep disturbance (35%), post-exertional malaise (30%), and neurosensory disturbance (30%). Sleep profiles and cognitive tests revealed distinctive impairments. Lower morning cortisol level and alterations in its diurnal rhythm were observed in the patients, and antibody and antigen measurements showed no evidence for acute infections by common viral or bacterial pathogens. These results highlight the urgent need of developing molecular diagnostic tests for ME/CFS. In addition, there was a striking similarity in symptoms between long COVID and ME/CFS, suggesting that studies on the mechanism and treatment of ME/CFS may help prevent and treat long COVID and vice versa.

## 1. Introduction

Myalgic encephalomyelitis/chronic fatigue syndrome (ME/CFS) is a chronic complex disease characterized by unrelenting fatigue, post-exertional malaise, sleep problems, cognitive impairment, and orthostatic intolerance [1]. This debilitating illness is known to affect between 836,000 and 2.5 million people in the United States alone [1,2,3], and the majority of the patients remain undiagnosed [1,4]. Patients often report symptoms started with viral infection [1,2,5]. Patients of ME/CFS have been found to be more functionally impaired than those with major diseases such as cancers, heart disease, and rheumatoid arthritis [6], and their prognosis remains poor [7,8]. Despite the severity of the clinical symptoms, the etiology and pathophysiology of the disease remain unclear. To date, there is neither a validated biomarker for diagnosis nor an FDA-approved drug available for treatment.

An estimated 25% of patients with ME/CFS are unfortunately severely affected and physically confined to their homes or beds [1,9,10]. These severely affected patients suffer from extreme daily fatigue, grievous impairments, and other debilitating symptoms. They often require in-home assistance and support adjusted explicitly to their needs [11,12]. However, severely affected patients are rarely studied [10,11], partially due to the difficulties accessing clinical care facilities. The personal account of an extremely severe patient is presented in this Special Issue [13]. To reduce the significant gap between the needs of severe patients and the healthcare they receive, there is an urgent need to better characterize these patients’ clinical conditions and discover the underlying biological abnormalities causing the symptoms [14]. In addition, as the condition worsens, the probability that biomarkers can be identified for the disease increases by studying severely ill patients.

Here we conducted a Severely Ill Patient Study (SIPS), which included a comprehensive examination of clinical symptoms and clinical lab tests of a cohort of severely ill patients and controls. First, questionnaires were administered to evaluate the patients’ quality of life, health status, and symptoms. Second, the patients’ daily activity, sleep profile, and cognitive capacity were monitored and examined to assess their symptoms objectively. Third, clinical laboratory testing and antigen & antibody tests against viral and bacterial pathogens were obtained. In addition, multiple omics studies are being conducted on the biological samples of these patients to identify molecular signatures of severe ME/CFS, and the results will be reported elsewhere. We have made the data and results available through a web-based data portal for the research community at https://endmecfs.stanford.edu.

## 2. Materials and Methods

### 2.1. Participants

Patients were identified for the study from an existing pool of homebound, and mostly bedbound, ME/CFS managed and diagnosed patients at the clinic sites of the investigators of the study and from those referred to the investigators to be eligible to participate in the study. ME/CFS clinicians at the study sites identified initially the potential subjects, who were most likely to be involved in this study, through the screening of medical records of these patients. Next, patients (age 18–70) were assessed for ME/CFS criteria online or by phone. They were consented if they met the International Consensus Criteria (ICC) for ME/CFS [15], were homebound (i.e., spending more than 14 h per day sedentary and in a reclined position as reported by patient or caregiver), and received a low score in physical status (i.e., SF-36 [16] physical functioning score and Karnofsky Performance Status Index [17] were both less than 70). They also must not fit the exclusion criteria. Consented patients were then provided with a FitBit device to confirm that they met the sedentary requirement. A complete blood count within the past 3–6 months was requested to verify anemia was not present (hematocrit > 34%). The detailed inclusion and exclusion criteria of the patients are listed in Section 2.1.1.

Healthy controls were evaluated for inclusion in the study based on meeting all inclusion criteria and not having any exclusion criteria (Section 2.1.2). These controls must be age 18 to 70, not carry a diagnosis of ME/CFS as defined by the ICC or active illness (acute or chronic), daily sedentary time ≤ 14 h, SF-36 physical functioning score ≥ 70, and without the conditions in the exclusion criteria.

All patient and control subjects were consented. Limited by available funding, 20 severely ill ME/CFS patients and 10 healthy controls were included in this study.

#### 2.1.1. Severely Ill ME/CFS Patients Inclusion and Exclusion Criteria

Inclusion Criteria

1. Age 18–70, inclusive;
2. Must carry a diagnosis of ME/CFS as defined by the ICC criteria;
3. Subjects must be homebound and spend >14 h per day sedentary and in a reclined position (measured by FitBit and patient/family report);
4. SF-36 physical functioning score < 70; and
5. Be able to provide informed consent.

Exclusion Criteria

1. Patients, age < 18 years or > 70 years;
2. Women who are pregnant;
3. Unable to understand informed consent; or
4. Patients with known HCT < 34 mg/dL.

#### 2.1.2. Healthy Control Inclusion and Exclusion Criteria

Inclusion Criteria

1. Age 18–70, inclusive;
2. Must not carry a diagnosis of ME/CFS as defined by the ICC criteria or active illness (acute or chronic);
3. Must be sedentary ≤ 14 h; and
4. SF-36 physical functioning score ≥ 70.

Exclusion Criteria

1. Patients, age < 18 years or > 70 years;
2. Women who are pregnant;
3. Unable to understand informed consent; or
4. Patients with a known HCT < 34 mg/dL.

### 2.2. Data Collection from Questionnaires

Questionnaires on Health Status and Quality of Life. The perceived health status and quality of life of the patients and the controls were evaluated by several sets of questionnaires, i.e., SF-36, Karnofsky Performance Status [17], Patient-Reported Outcomes Measurement Information System (PROMIS) instruments [18,19] (including Fatigue, Pain Behavior, Pain Interference, Sleep Disturbance, and Sleep-Related Impairment), Pittsburgh Sleep Quality Index (PSQI) [20], and a questionnaire on Restless Leg Syndrome (RLS) [21].

Evaluation of Common Symptoms in Patients. Patients were evaluated using a set of 7 symptoms-related questions which covered the common symptoms mentioned in ICC [15] and IOM [1]. The text-based answers were transformed to 79 numerical or categorical measurements, indicating if a subject had a particular impairment/symptom or quantifying the degree of the impairment/symptom. These were then grouped into 12 symptomatic categories, which represented the 5 core symptoms of ME/CFS in the 2015 IOM diagnostic criteria [1] (i.e., fatigue, post-exertional malaise, sleep disturbance, cognitive impairment, and orthostatic intolerance) and 7 additional common accompanying symptoms mentioned in the IOM or ICC criteria (i.e., pain, neurosensory disturbance, flu-like symptoms and/or susceptibility to viral infections, gastrointestinal tract impairment, loss of thermostatic stability and/or intolerance of extremes of temperature, respiratory impairments, and genitourinary impairments). In addition, the top 3 most troublesome symptoms of each patient were recorded.

### 2.3. Data Collection of Patient Activity, Sleep Monitoring, and Cognitive Tests

**Activity Monitoring.** Patients were provided with a Charge HR (FitBit, Inc., San Francisco, CA, USA) for two weeks. This device documented patient activity and continual heart rate to confirm that patients met the sedentary requirement. The measurements, including Active Minutes, Sleep Duration, Sleep Score, Sleep Time, Calories Burned, Distance, Floors, Steps and Resting Heart Rate, were retrieved with the R package fitbitScraper and summarized to the daily average.

Sleep Monitoring. Patients underwent an overnight sleep profiler study. The non-invasive sleep monitor was the Sleep Profiler [22] from Advanced Brain Monitoring (Carlsbad, CA, USA) and consisted of a 3-lead EEG, snore (audio) detector, activity/motion detector, and an eye movement detector. The overnight EEG and other signals were reviewed by the study staff. Thirty-five measurements on the sleep architecture & continuity (e.g., total sleep time, sleep efficiency and sleep latency) and cardio-respiratory signals (e.g., pulse rate and snoring) were analyzed and compared with the established normative ranges [23]. Sleep abnormalities were then identified and compared with sleep EEG biomarkers that were associated with chronic health conditions or neurological diseases [24].

Cognitive Tests and Extended EEG. WebNeuro Tests (Brain Resource Group, San Francisco, CA, USA) [25] were utilized to evaluate the cognitive performance of the patients and the controls. Four types of cognitive abilities (i.e., attention, maze, memory, and identifying emotions) were evaluated. The results were scored against a cohort of normative subjects in the Brain Resource International Database (BRID) [26]. The normalized scores (Z-scores) and the corresponding implications (e.g., Z-score £-2 implies clinical significance) were reported in WebNeuro Report (Version: WebNeuro Short 3.1.5). The clinical/research grade EEG device was a 24 channel Stat X24 also from Advanced Brain Monitoring. Twenty electrodes on the head were monitored in this study. Extended EEG monitoring was combined with the cognitive test for the patients and controls. Before or after the test, 15 min of EEG was monitored as the standard control. During the four tests: attention, maze, memory and emotion, EEG was monitored simultaneously.

### 2.4. Clinical Lab Tests

For clinical tests, a maximum of 160 ml of blood was collected from each subject for clinical tests. Blood samples were collected from all ME/CFS subjects when a research team visited the subject’s home and performed the physical exam. Samples were collected from all healthy control subjects during their visit to the clinic. Urine over 24-h and saliva specimens were also collected from the subjects. To reduce the variability of the test results across the study population, all samples were collected on the same day during the patient’s appointment. The samples were shipped to routine and specialty clinical labs. All clinical laboratories are CLIA approved.

The tests were chosen based on results from previous studies on ME/CFS (Table S1). These included complete blood count with differential, comprehensive metabolic panel, standard lipid panel, acylcarnitine profile, urinalysis of organic acids, hormones (including cortisol, thyroid-stimulating hormone/thyroid hormones (TSH/T3/T4), follicle-stimulating hormone and luteinizing hormone (FSH/LH), testosterone, estrogen, and arginine vasopressin), vitamins (B7/biotin, B12/folate, D, methylmalonic acid), selected chemistry analytes and disease biomarkers, lymphocyte subsets, and natural killer cell function. Salivary cortisol monitoring was tested for each subject at four time points of the day: 30 min after morning awakening, noon, afternoon, and night. All these tests were performed by Quest Diagnostics (Secaucus, NJ, USA).

### 2.5. Tests of Antibodies and Antigens against Pathogens

Also performed were tests on antibodies and antigens against viral and bacterial pathogens (Table S2). The tests of IgG and IgM antibodies against viruses were conducted at Quest Diagnostics, which included Herpes simplex virus 1 and 2 (HSV1/2, HHV1/2), Epstein-Barr Virus (EBV, HHV4), *Cytomegalovirus* (CMV, HHV5), Human Herpesvirus 6 and 7 (HHV6/7), and Primate *Erythroparvovirus* 1 (Parvovirus B19, B19).

Lyme disease antibody tests were performed at Quest Diagnostics, which included IgG and IgM antibody tests and the Western blot [27,28]. For the Western blot, *Borrelia burgdorferi* IgM was considered positive if two of the three bands were present; IgG was considered positive if five of the 10 bands were present [27]. In addition, Ceres Nanotrap antigen tests (Ceres Nanosciences, Manassas, VA, USA) were performed to detect *Borrelia* Outer surface protein A (OspA) antigen [29]. *Mycoplasma pneumoniae* IgG and IgM antibodies were tested by Quest Diagnostics. *Bartonella* tests were performed at Galaxy Diagnostics (Research Triangle Park, NC, USA), which included a PCR test of *Bartonella* species of the whole blood, serum and blood cultures at 8 days, 14 days, and 21 days. In addition, immunofluorescence assay (IFA) was used for the IgG of *Bartonella henselae* and *Bartonella quintana* and results with titers of ≥1:256 were considered to be positive for the analysis [30].

The biological samples collected were also archived for further omics studies of genes, proteins, metabolites, and microbes present in severely ill ME/CFS patients.

### 2.6. Data Analysis

To compare the quality of life and the patient-reported health status between SIPS patients and controls, Wilcoxon signed-rank test was used. To visualize the closeness/distance of SF-36 among SIPS samples, general CFS, and other related medical conditions, tSNE was utilized (implemented in the R package Rtsne) to project the SF-36 scores to two dimensions.

To quantify the severity and frequency of the 12 symptomatic categories in SIPS, we operationally defined a burden score for each category that could summarize the 95 symptomatic measurements from all questionnaires. We first unified the ranges and directions of the measurements. After the standardization, all the measurements ranged from 0 to 1, and the higher value indicated the worsening of the symptom. Specifically, for quantitative phenotypes, we used the formula x−min(x)/max(x)−min(x) to re-scale the values of each measurement and reversed its direction if the average of healthy controls was larger than the average of SIPS patients. For binary phenotypes where 1 indicated having the symptoms, the values were weighted by the frequencies of the symptoms in the patients. We calculated the burden score of each symptomatic category by averaging the standardized measurements assigned to the category. The burden scores were visualized with the R package heatmaply, and the individuals were hierarchically clustered by their Euclidean distances.

For Fitbit measurements and cognitive test STEN (Standard Tens) scores, Student’s *t*-test was performed to test if there was a significant difference between SIPS patients and controls. One-sided Fisher’s exact test was performed to test if there is a significantly higher number of patients with a clinically significant low STEN score for the four types of cognitive abilities.

For each of the clinical tests, where the diverse raw values hardly followed a normal distribution, we performed Box-Cox transformation to fit the values from health controls into a normal distribution. A bootstrap *t*-test was also performed on the clinical tests to generate the *p*-values, and FDRs were also calculated.

The prevalence of the symptoms remaining after six months in long COVID reported in a recent study [31] was retrieved from the Appendix and Figure 11a of the article and compared with the correspondent symptoms in the SIPS patients.

All the analyses and visualization were performed with the R program.

## 3. Results

Results include Patient-reported health status and symptoms (Section 3.1), Activity, sleep monitoring, and cognitive tests (Section 3.2), Clinical laboratory testing (Section 3.3), and Antigen and antibody tests against viral and bacterial pathogens (Section 3.4). All results described below were based on data from the entire cohort unless otherwise indicated. The data and results are available through a web-based data portal at https://endmecfs.stanford.edu.

### 3.1. Patient-Reported Health Status and Symptoms of the Severely Ill

#### 3.1.1. Demographics and Quality of Life of the Patients

The demographics of the subjects of the study are shown in Table 1. In the SIPS patients, the duration of the illness ranged from 2.4 years to 50 years, with a mean of 14.5 years. While all the patients were homebound, half of them required considerable assistance and frequent medical care, and 35% were disabled and needed special care and assistance, as indicated by the Karnofsky scale. 

**Figure 1:**
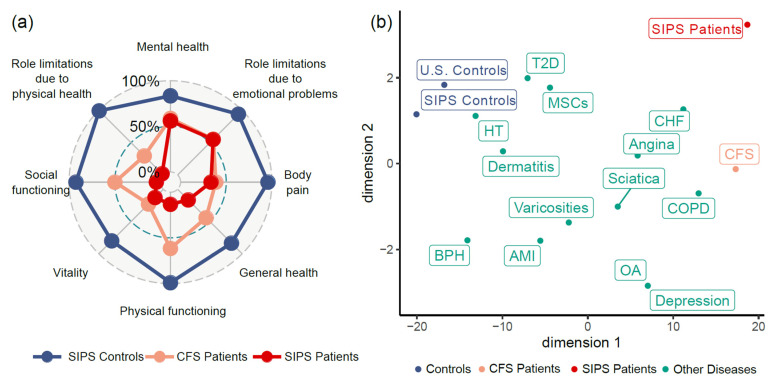
Comparison of the Quality of Life of Severely Ill myalgic encephalomyelitis/chronic fatigue syndrome (ME/CFS) and Other Major Diseases. (**a**) SF-36 scores of Severely Ill Patient Study (SIPS) patients, general CFS patients, and healthy controls. Compared with the general CFS patient population, scores on physical functioning (PF), role limitations due to physical health (RP), general health (GH), vitality/energy/fatigue (VT), and social functioning (SF) were significantly lower. (**b**) tSNE of SF-36 scores of SIPS, general CFS, and other medical conditions. T2D—type II diabetes, HT—hypertension, CHF—congestive heart failure, COPD—chronic obstructive pulmonary disease, MSCs—musculoskeletal complaints, BPH—benign prostatic hyperplasia, AMI—anterior myocardial infarction, and OA—Osteoarthritis. The quality-of-life scores of SIPS patients were clearly separated from that of controls, being most positively correlated with congestive heart failure (CHF) and most negatively correlated with clinical depression.

The SF-36 results showed that SIPS patients had significantly lower scores in comparison with healthy controls (Table 1 and Figure 1a). In particular, scores on physical functioning (PF), role limitations due to physical health (RP), general health (GH), vitality/energy/fatigue (VT), and social functioning (SF) were extremely low, with each less than 20. As shown in Figure 1a, comparing to the scores of the general patients of ME/CFS [32], each of these five scales was further lowered significantly in the SIPS patients. This is also consistent with other published studies on the quality of life of ME/CFS. For example, in the phase 3 trial of rituximab (RituxME), the average PF score was >30 for the patients (35.2 ± 21.9 and 32.5 ± 19.1 for the treated and placebo groups, respectively) [33], while in this study, the PF score was <15 in the severely ill patients (13.3 ± 12.8). Our results suggested that severe illness had greatly reduced the quality of life of these severely ill patients, even further than the general ME/CFS patient population. 

On the other hand, the role limitations due to emotional problems (RE) was much less impacted, followed by mental health/emotional well-being (MH), in SIPS patients, which appeared to be similar to general ME/CFS (Figure 1a).

We next compared the SF-36 scores of SIPS with that of other medical conditions in the USA [34]. The results showed that the SF-36 scores of SIPS were well separated from the general U.S. population as well as other medical conditions (Figure 1b). Compared to other major diseases, the severely ill ME/CFS patients had lower scores in six of the eight scales, except RE and MH (Figure S1a). In addition, among these medical conditions, the quality-of-life scores of the SIPS patients were most positively correlated with Congestive Heart Failure (*r* = 0.63) and most negatively correlated with Clinical depression (*r* = −0.33) (Figure S1b).

#### 3.1.2. Patient-Reported Health Status

Several sets of questionnaires were administered to evaluate the health status of the patients. Five PROMIS instruments were utilized, which provided measures of physical, mental, and social well–being from the patient perspective. As shown in Table 2, comparing with the controls, the severely ill patients reported significant fatigue, sleep disturbance, sleep-related impairment, the experience of pain (pain behavior), and interference of pain on activities (pain interference).

Similarly, the analysis of the results of the Pittsburg Sleep Quality Index (PSQI) showed significantly lower sleep quality, more sleep disturbances, and worse Global PSQI in the patients compared to the controls. In addition, 4 of the patients (20%) had probable Restless Leg Syndrome (RLS).

#### 3.1.3. Evaluation of the Common Symptoms in the Patients

Data on specific symptoms known to be correlated with ME/CFS [1,15] were obtained using a standardized questionnaire. The results are shown in Figure S2a, indicating whether a patient or a control had a particular impairment or the degree of the impairment. Figure S2b shows a hierarchical clustering of these symptoms between the patients, where symptoms related to sleep disturbance and symptoms related to pain clustered together. These individual symptoms were then grouped into 12 symptomatic categories, which were mentioned in the IOM and ICC descriptions of ME/CFS. One of the extremely ill patients was not able to complete the questionnaire and was not included in the downstream analysis.

**Figure 2:**
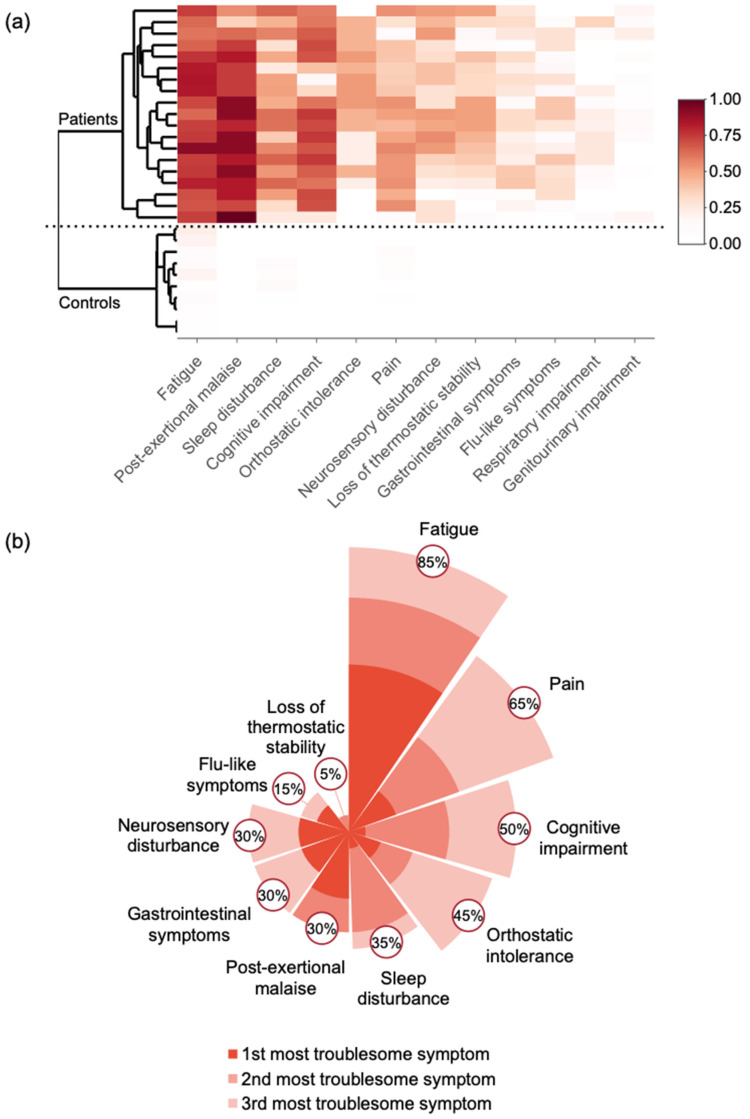
Common Symptoms in Severe ME/CFS Patients. (**a**) Similarity and variation of the symptoms of the SIPS patients and (**b**) the top three most troublesome symptoms of the SIPS Patients.

As shown in Figure 2a, all the patients had fatigue, sleep disturbance, and post-exertional malaise, and had either cognitive impairment (19/19 or 100%) or orthostatic intolerance (15/19 or 79%), or both. Therefore, all the patients met the IOM ME/CFS diagnosis criteria. Additional symptoms include pain, neurosensory disturbance, flu-like symptoms and/or susceptibility to viral infections, gastrointestinal tract impairments, Genitourinary impairment, and Respiratory impairment. Notably, 100% of the patients (19/19) suffer from the presence of significant pain and 89% (17/19) had sensitivity to light, noise, vibration, odor, taste, and touch (Figure S2a).

We next looked at the top 3 most troublesome symptoms of the severe patients (Figure 2b). The symptoms reported by the patients were fatigue (85%), pain (65%), cognitive impairment (50%), orthostatic intolerance (45%), sleep disturbance (35%), post-exertional malaise (30%), neurosensory disturbance (30%), GI tract impairment (30%), flu-like symptoms (15%), and loss of thermostatic stability (5%). Fatigue and post-exertional malaise were ranked most commonly as the top troublesome symptom by 50% and 20% of the patients, respectively.

### 3.2. Activity, Sleep Monitoring, and Cognitive Tests of the Severe ME/CFS Patients

The severely ill patients in the study were homebound and spending more than 14 h per day sedentary and in a reclined position as reported by the patient or caregiver. To objectively monitor the physical activities of the severely ill patients, patients were provided with a FitBit device. The median daily steps taken by the SIPS patients was 912, which was significantly lower than that of the healthy controls as well as the reported values from previous studies of the U.S. population [35,36], and similar results were seen on the daily distance, the number of floors taken, and calories burned. These results confirmed that the mobility of the patients was severely limited by the disease.

**Figure 3:**
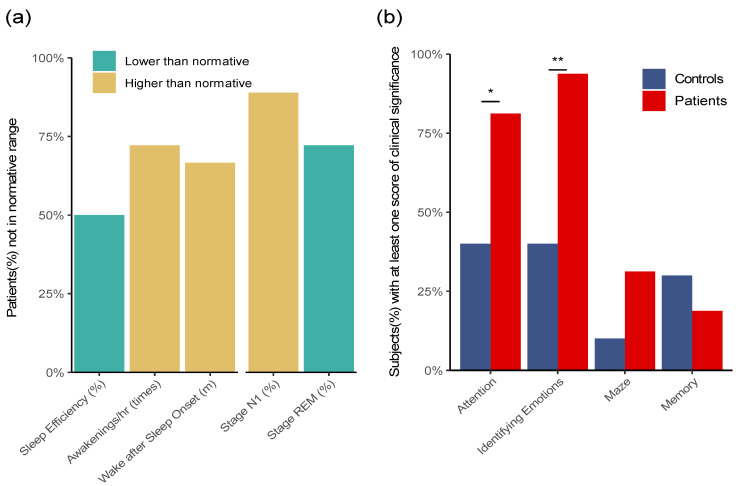
Sleep Monitoring and Cognitive Tests of the Severe ME/CFS Patients. (**a**) Five parameters in the overnight monitoring by Sleep Profiler where the values in ≥50% of the patients were consistently out of the normal ranges. These include lower sleep efficiency, more frequent awakenings per hour (>30 s), longer time of wake after sleep onset, a higher percentage of Stage N1, and a lower percentage of Stage R (REM). (**b**) Comparison between the patients and controls in each of the four sections of cognitive tests. The Y-axis represents the percentage of subjects that were identified as severe/deficit impairment. The patients compared with the controls showed significantly more problems in identifying emotions (94% of the patients vs. 40% of the controls, *p* = 0.005), as well as more attention problems (81% of the patients vs. 40% of the controls, *p* = 0.046).

Sleep-related problems, such as insomnia, sleep disturbances, and unrefreshing sleep, are among the core symptoms of ME/CFS [37,38]. Overnight sleep of the patients was monitored by a non-invasive Sleep Profiler (Advanced Brain Monitoring). Sleep time and efficiency, sleep architecture, latencies, and continuity, snoring, and cardio were reviewed by the study staff and analyzed comparing with the established normative ranges [23]. Five parameters were identified in the sleep profile where in more than 50% of the patients, the measurements were consistently out of the normal range, that is, either exclusively below the lower limit or above the higher limit of the normal range. Figure 3a shows these five parameters and the percentages of patients whose parameters fell out of the normal ranges. Among the severely ill patients, 75% had an abnormally higher number of awakenings (Awakening/hr ≥ 30 s), 65% had abnormally longer wake time after sleep onset (Wake after Sleep Onset), and 50% had sleep efficiency (Sleep Efficiency) below the normal range. Further, the EEG profile revealed that in 70% of the patients, the percentages of Stage R (REM) were below the normal range, and conversely, in 90% of the patients, the percentages of Stage N1 were above the normal range. The observed high percentages of Stage N1 and low percentages of Stage R were consistent with the frequent awakenings during the sleep observed in these patients [39].

Cognitive abnormalities are prevalent in ME/CFS, which include poor attention and concentration, slow information processing, and impaired memory registration and consolidation [40,41,42]. The cognitive performance of the patients and controls was evaluated using WebNeuro Tests (Brain Resource Group). Four types of cognitive abilities—attention, maze, memory, and identifying emotions—were evaluated and compared with established normal ranges [43]. When comparing the patients with controls, the most significant difference is the higher number of the SIPS patients who had issues in identifying emotions, where their scores were outside of the normal range (94% of the patients vs. 40% of the controls, *p* = 0.005). In particular, the reaction time of the patients was significantly longer than that of the controls for both happiness and anger (*p* = 0.015 and 0.007, respectively). In addition, the patients showed more attention problems than the controls (81% of the patients vs. 40% of the controls, *p* = 0.043). In contrast, the SIPS patients did not show a significant difference in the scores for memory and maze. Similarly, we did not identify any consistent difference between the patients and the controls in the EEG signal monitored taken during the cognitive tests, which potentially were due to the heterogeneity in the data acquired.

### 3.3. Results of Clinical Laboratory Testing

To systematically evaluate whether clinically recognized biomarkers show the difference between severe ME/CFS and healthy controls, an extensive set of clinical laboratory tests were performed on the blood, urine, and saliva samples in this study.

**Figure 4:**
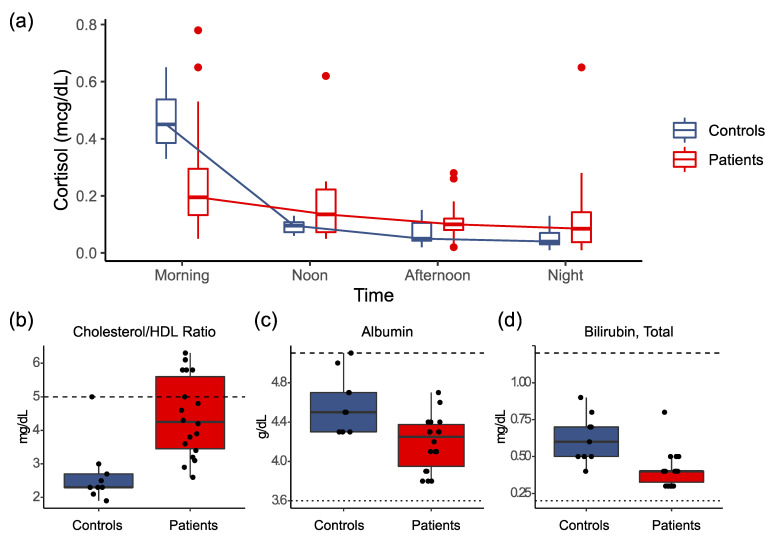
Clinical Lab Test Results Significantly Different between Severe ME/CFS and Controls. (**a**) Results of 4-point salivary cortisol upon wakening, at noon, afternoon, and night. The severe patients demonstrated significantly lower salivary cortisol concentrations in the morning and a significant flattening of the diurnal cortisol profile. (**b**–**d**) Results of a significantly higher level of cholesterol/HDL ratio (**b**), lower level of albumin, (**c**) and lower total bilirubin, (**d**) in the blood of the patients than of the controls.

The most significant difference between severe ME/CFS and the controls came from the 4-point salivary cortisol levels, which were tested upon wakening, at noon, afternoon, and night. In healthy individuals, the cortisol level increases upon wakening and steadily decreases throughout the day. As shown in Figure 4a, the severe patients showed significantly lower salivary cortisol concentrations in the morning, where the median levels were 0.20 mcg/dL and 0.45 mcg/dL in the patients and controls, respectively (*p* = 0.002). In addition, there was a significant reduction of the decrease in the cortisol level over the day in the patients compared to the controls: the mean coefficient (slope) of the cortisol level (in log scale) over time (in hours) was −0.059 in the patients and −0.156 in the controls (*p* = 0.003).

Figure 4b–d show additional results significantly different between the severely ill patients and the controls (FDR < 0.1). These include a higher level of cholesterol/HDL ratio (b), lower level of albumin (c), and lower total bilirubin (d) in the blood of the patients than of the controls. On the other hand, no significant differences were observed in the rest of the lab tests, including CBC with DIFF/PLT, Lymphocyte Subsets, Natural Killer Cell function, Comprehensive Metabolic Panel, Standard Lipid Panel, Acylcarnitine Profile, Urinalysis of organic acids, hormones (TSH/T3/T4, FSH/LH, testosterone, estrogen, AVP)), vitamins (B7/biotin, B12/folate, D, Methylmalonic Acid), and selected chemistry analytes and disease biomarkers. The results are shown in Table S1.

### 3.4. Tests on Antigens and Antibodies against Viral and Bacterial Pathogens

Since ME/CFS patients often report symptoms started with a viral infection, we tested in the patients and the controls antibodies and antigens of a set of common pathogens. These included IgG and IgM antibodies against human herpesvirus 6 and 7 (HHV-6/7), herpes simplex virus 1 and 2 (HSV-1/2 or HHV-1/2), Epstein-Barr virus (EBV or HHV-4), *Cytomegalovirus* (CMV or HHV-5), and parvovirus B19. In addition, tests were performed to detect antigens and antibodies of *Borrelia burgdorferi*, *Bartonella* species, and *Mycoplasma pneumoniae*.

As shown in Table 3, there was no significant difference detected between the severely ill patients and the healthy controls in the tests performed. The percentages of samples identified as positive in each test were similar for each of the antibody and antigen tests of viral and bacterial pathogens. More detailed information can be found in Table S2.

IgM antibodies against the common viruses were either not detected or detected positive in very few of the patients and the controls at the same percentage. These include HHV-6 (1/18 in patients vs. 0/9 in controls), EBV (0/18 in patients vs. 0/9 in controls), B19 (0/18 in patients vs. 0/9 in controls), CMV (1/18 in patients vs. 0/9 in controls), and HSV-1/2 (2/17 in patients vs. 1/9 in controls). On the other hand, IgG antibodies were detected in large percentages of both the patients and the controls for these viruses, which included, in patients vs. in controls, HHV-6 (19/19 vs. 9/9), EBV (VCA: 17/18 vs. 9/9, and EBNA: 16/19 vs. 8/8), parvovirus B19 (14/18 vs. 6/9), CMV (9/18 vs. 4/9), and HSV-1 and HSV-2 (6/18 vs. 2/9 and 6/18 vs. 1/9, respectively).

Similarly, few bacterial antigen or IgM tests were positive in patients (0/18 for *Borrelia burgdorferi* IgM, 2/18 for *Borrelia* OspA, 1/19 for *Mycoplasma pneumoniae* IgM, and 0/20 for PCR of *Bartonella* DNA in blood, serum, and culture) without any significant difference comparing to the results of the controls (0/9 for *Borrelia burgdorferi* IgM, 1/10 for *Borrelia* OspA, 0/10 for *Mycoplasma pneumoniae* IgM, and 0/9 for PCR of *Bartonella* Species in Blood, Serum, and Culture). In the same samples, IgG antibodies were detected at the same rate in patients vs. in controls (0/18 vs. 0/9 for *Borrelia Burgdorferi*, 13/18 vs. 6/7 for *Mycoplasma Pneumoniae*, 11/20 vs. 4/9 for *Bartonella Henselae*, and 7/20 vs. 4/9 for *Bartonella Quintana*).

## 4. Discussion

ME/CFS significantly reduces the quality of life of patients [6,44,45,46], and the severe cases studied here present a picture of a systematically debilitating disease. Severely affected patients who were homebound and mostly bedbound suffer from a greater reduction of their quality of life compared to other major chronic diseases as well as the general ME/CFS population. While physical functioning, energy/fatigue, and related functioning were extremely low in these patients, emotional well-being was clearly less impacted-a clear distinction from the frequent misdiagnosis of clinical depression in these patients.

The SIPS patients had all the core symptoms in the IOM criteria [1] and other symptoms such as pain and neurosensory disturbance, consistent with previous reports [1,11,12]. The most troublesome symptoms were fatigue (85%), pain (65%), cognitive impairment (50%), orthostatic intolerance (45%), sleep disturbance (35%), post-exertional malaise (30%), neurosensory disturbance (30%), GI tract impairment (30%). Pharmacological and non-pharmacological approaches to the relief of these symptoms could help individual patients manage this disease, since there are no treatments currently approved for ME/CFS [47,48].

Sleep disorders and cognitive impairments are core symptoms of ME/CFS [37,38,40,41,42]. Non-invasive sleep monitoring revealed that the majority of the severely ill patients had an abnormally high number of awakenings, abnormally long wake time after sleep onset, and sleep efficiency below the normal range, which are consistent with the high percentages of Stage N1 and low percentages of Stage R (REM) observed in their EEG profiles [39]. Cognitive tests showed significant differences in the severely ill patients in identifying emotions and having attention problems, while there was no difference in the maze and memory tests between the patients and the controls. Impairment of divided attention has been reported previously in ME/CFS patients [40,49], and our results are consistent with the hypothesis that the difficulty in divided attention may contribute significantly to the cognitive problems in ME/CFS. Further studies using sophisticated methodologies are essential to better characterize and understand the sleep and cognitive disorders in ME/CFS.

Currently, there is no diagnostic test for ME/CFS, and laboratory tests are primarily used in differential diagnosis to identify alternative conditions and comorbidities [14,47,50]. Here we evaluated an extensive set of clinical lab tests in blood, urine, and saliva samples. Between the severely ill patients and the controls, the most significant difference observed was lower salivary cortisol concentrations in the morning and the flattening of the daily cortisol profile in the patients, consistent with previously reported observations of the alterations in diurnal salivary cortisol rhythm in ME/CFS [51,52]. Other tests conducted did not show noticeable significance. While we did not perform all the recommended testing by the US ME/CFS Clinician Coalition [4,50], these lab results re-confirm the limitations of the standard laboratory test battery in ME/CFS and highlight the urgent need of developing new diagnostic tests for the disease [1,14]. For instance, lower-than-normal circulating blood volume could be associated with orthostatic intolerance seen in the severely ill patients, which would be worthwhile measuring [53].

Previous studies showed that in many ME/CFS patients, the ‘sudden onset’ of the disease appears to be a viral infection [2,5,54,55]. Therefore, we tested antibodies and antigens of a set of common viral and bacterial pathogens. The results showed no evidence for acute infections by the tested pathogens in the patients, while as expected, large percentages of both the patients and the controls had been exposed to some of these common viral or bacterial pathogens. Enteroviruses were proposed as a cause of ME/CFS [56,57] but were not tested in this study. Also, it is worth noting that certain pathogens are neurotropic and evidence of central nervous system (CNS) infection is not always revealed by serologic studies of blood. Further analysis of autoantibodies and detections of pathogens (e.g., by sequencing) in the relevant tissues, such as in the cerebrospinal fluid (CSF), will likely provide new insights into the link between pathogen exposure and ME/CFS.

The biological samples collected on the severely ill patients and the healthy controls are being further analyzed in multiple omics studies to identify signatures in genes, proteins, metabolites, heavy metals, and microbes of severe ME/CFS and the associated clinical symptoms.

**Figure 5:**
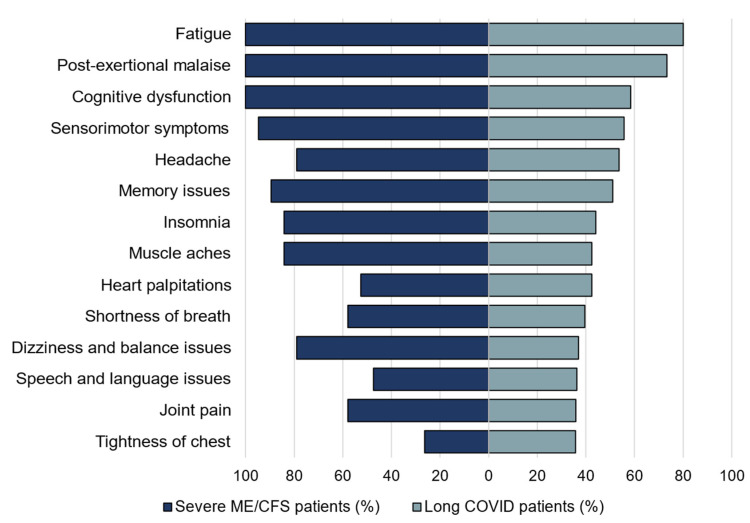
Comparison of the symptoms reported in the long COVID patients after 6 months with those in the severely ill ME/CFS patients. The symptoms are ranked based on the frequencies reported in the long COVID patients. The frequencies of these symptoms in the severely ill ME/CFS are similar to those reported in the long COVID.

Post-COVID conditions (long COVID, Post-Acute Sequelae of SARS-CoV-2 infection (PASC)), are affecting an increasingly large number of people worldwide, where patients suffer from prolonged fatigue and other symptoms [58,59,60]. A recent study [31] of 3762 confirmed or suspected COVID patients from 56 countries showed that the time to recovery in most patients exceeded 7 months, where the majority of the patients had multiple symptoms related to ME/CFS. Therefore, we compared the frequencies of the symptoms remaining after six months in the long COVID patients with those of the severely ill ME/CFS patients, and the results showed a striking similarity (Figure 5). This underscores the value of research to understand the mechanisms of ME/CFS for efforts to treat and prevent long COVID and other debilitating postviral conditions, which together affect millions in the United States alone [14,61].

## Figures and Tables

**Table 1:** Demographics and quality of life of severe myalgic encephalomyelitis/chronic fatigue syndrome (ME/CFS) patients and healthy controls.

	Patients (*N* = 20)	Controls (*N* = 10)	*p*-Value ^1^
Age (years; mean ± s.d.)	47.4 ± 11.6	46.8 ± 9.2	0.552
Sex (% female)	65.0%	60.0%	0.813
BMI (kg/m^2^; mean ± s.d.)	25.4 ± 6.8	22.0 ± 3.1	0.224
Duration of illness (years; mean ± s.d.)	14.5 ± 11.8	0.0 ± 0.0	
*Karnofsky Performance status index (%)*			<0.001
30: Severely disabled; hospital admission is indicated, although death is not imminent.	5.0%	0.0%	
40: Disabled; requires special care and assistance.	30.0%	0.0%	
50: Require considerable assistance and frequent medical care.	15.0%	0.0%	
60: Require occasional assistance, but is able to care for most personal needs.	50.0%	0.0%	
100. Normal; no complaints; no evidence of disease.	0.0%	100.0%	
*Quality of life (SF-36 scores; mean ± s.d.)*			
PF: Physical functioning	13.3 ± 12.8	99.0 ± 2.1	<0.001
RP: Role limitations due to physical health	1.9 ± 6.1	99.4 ± 2.0	<0.001
RE: Role limitations due to emotional problems	55.0 ± 45.9	94.2 ± 9.7	0.037
VT: Vitality/Energy/Fatigue	12.8 ± 19.3	80.0 ± 14.7	<0.001
MH: Mental health/Emotional well-being	56.0 ± 25.8	83.5 ± 16.0	0.005
SF: Social functioning	4.4 ± 12.4	92.5 ± 13.4	<0.001
BP: Body pain	33.4 ± 26.2	95.8 ± 7.6	<0.001
GH: General health	16.5 ± 7.3	83.5 ± 15.1	<0.001

^1^ Wilcoxon signed-rank test.

**Table 2:** Comparison of patient-reported health status between severe ME/CFS patients and healthy controls.

	Patients	Controls	*p*-Value ^1^
*PROMIS Instruments (T-score; mean ± s.d.)*			
Fatigue	75.2 ± 5.9	41.8 ± 9.6	<0.001
Sleep disturbance	64.5 ± 7.5	39.7 ± 7.4	<0.001
Sleep-related impairment	65.4 ± 7.4	37.5 ± 8.4	<0.001
Pain interference	67.0 ± 10.1	44.5 ± 4.8	0.003
Pain behavior	60.6 ± 8.9	42.4 ± 11.5	0.004
*Pittsburgh Sleep Quality Index (mean ± s.d.)*			
Sleep quality	2.1 ± 0.7	0.0 ± 0.0	<0.001
Sleep latency	2.1 ± 1.3	1.0 ± 0.8	0.093
Sleep duration	0.4 ± 0.8	0.3 ± 0.5	0.814
Habitual sleep efficiency	1.6 ± 1.3	0.0 ± 0.0	0.019
Sleep disturbances	1.9 ± 1.0	0.3 ± 0.5	0.009
Use of sleeping medications	2.2 ± 1.1	0.0 ± 0.0	<0.001
Daytime dysfunction	1.9 ± 1.3	0.8 ± 0.5	0.144
Global PSQI score	11.9 ± 3.4	2.3 ± 1.7	0.003
*Restless Legs Syndrome (RLS; %)*			
Probable RLS	23.5% (4/17)	0.0% (0/4)	

^1^ Wilcoxon signed-rank test.

**Table 3:** Tests on antibodies and antigens.

Viruses-Antibody Tests	Patients Positive/Total	Controls Positive/Total	p-Value ^1^
*Cytomegalovirus* (IgG)	9/18	4/9	1
*Cytomegalovirus* (IgM)	1/18	0/9	1
Parvovirus B19 (IgG)	14/18	6/9	0.653
Parvovirus B19 (IgM)	0/18	0/9	1
Epstein-Barr Virus Early Antigen D (IgG)	2/18	2/9	0.582
Epstein-Barr Virus Viral Capsid Antigen (IgM)	0/18	0/9	1
Epstein-Barr Virus Viral Capsid Antigen (IgG)	17/18	9/9	1
Epstein-Barr Virus Nuclear Antigen (IgG)	16/19	8/8	0.532
Herpesvirus 6 (IgG)	19/19	9/9	1
Herpesvirus 6 (IgM)	1/18	0/9	1
Herpesvirus 7 (IgG)	0/19	0/9	1
Herpesvirus 7 (IgM)	0/18	0/9	1
Herpes Simplex Virus 1 (IgG)	6/18	2/9	0.676
Herpes Simplex Virus 2 (IgG)	6/18	1/9	0.363
Herpes Simplex Virus 1/2 (IgM)	2/18	1/9	1
***Bacteria-Antigen and Antibody Tests***	**Positive/Total**	**Positive/Total**	***p*-Value ^1^**
*Borrelia*-Ceres Nanotrap Lyme Antigen Test	2/18	1/10	1
Lyme Disease Ab with Reflex to Blot (IgG)	0/18	0/9	1
Lyme Disease Ab with Reflex to Blot (IgM)	0/18	0/9	1
*Borrelia burgdorferi* (IgG)	0/18	0/9	1
*Borrelia burgdorferi* (IgM)	0/18	0/9	1
*Mycoplasma pneumoniae* (IgG)	13/18	6/7	0.637
*Mycoplasma pneumoniae* (IgM)	1/19	0/9	1
Bartonella DNA-(Blood, Serum, and Culture)	0/20	0/9	1
*Bartonella henselae* (IgG)	18/20	8/9	1
*Bartonella quintana* (IgG)	17/20	7/9	0.633
***Immunoglobulin G Subclasses Panel***	**Low/Total**	**Low/Total**	***p*-Value ^1^**
Immunoglobulin G, subclass 1	1/19	0/9	1
Immunoglobulin G, subclass 2	0/19	2/9	0.095
Immunoglobulin G, subclass 3	3/19	0/9	0.530
Immunoglobulin G, subclass 4	1/19	2/9	0.234
Immunoglobulin G, serum	0/19	0/9	1

^1^ Fisher’s exact test.

## Data Availability

Deidentified data and results are available through a web-based data portal at https://endmecfs.stanford.edu.

## Supplementary Materials

The following are available online at https://www.mdpi.com/article/10.3390/healthcare9101290/s1, Figure S1: Comparison of the Quality of Life of Severely Ill ME/CFS and Other Major Diseases, Figure S2: Similarities and Differences in Clinical Symptoms across 20 SIPS Patients. Table S1: List of Clinical Laboratory Tests. Table S2: List of Antibody and Antigen Tests of Viral and Bacterial Pathogens.
