# Supplementary material for: A Comprehensive Examination of Severely Ill ME/CFS Patients
Source: Healthcare (Basel). 2021 Sep 29;9(10):1290. doi: 10.3390/healthcare9101290 (PMC8535418; doi:10.3390/healthcare9101290)
Supplement: Supplementary file 1 [file healthcare-09-01290-s001.zip › healthcare-1385639_supplementary figures.pdf]

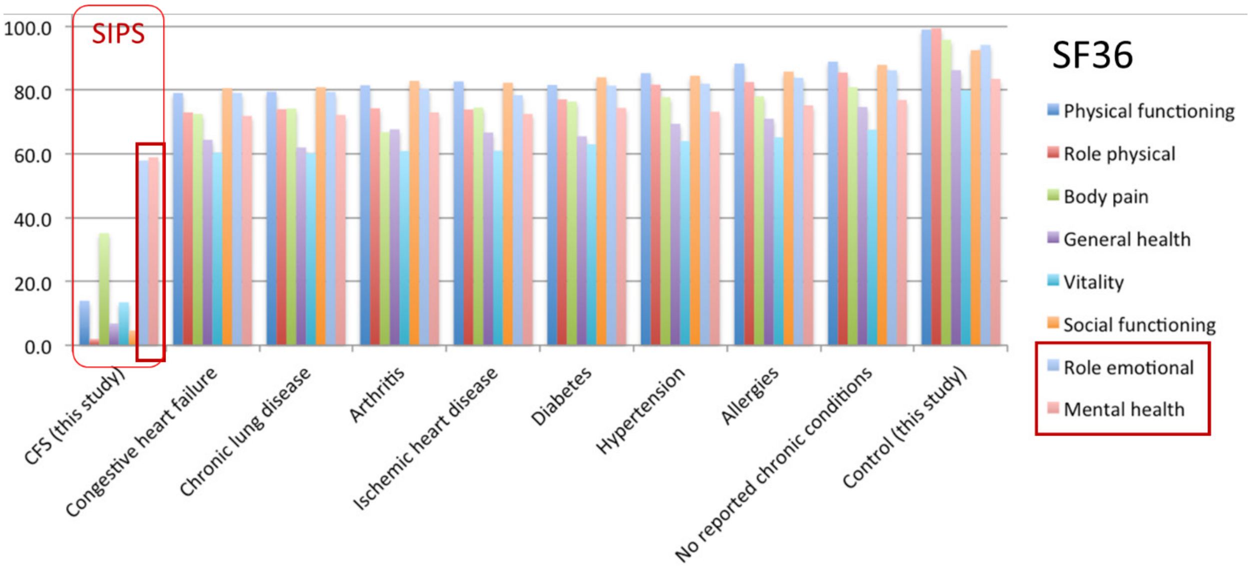

(a)

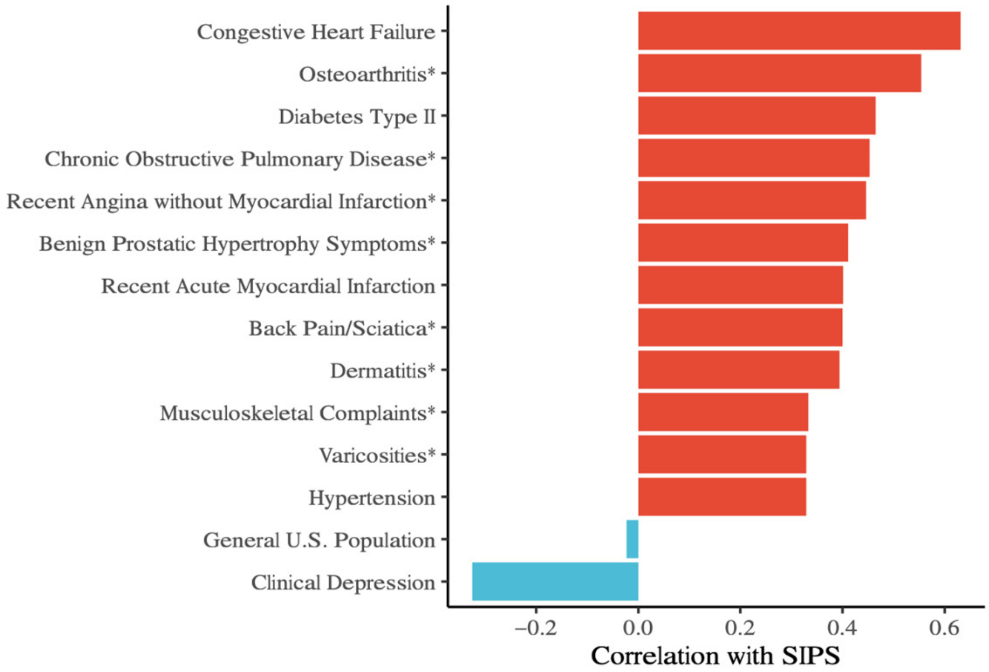

(b)

Figure S1 Comparison of the Quality of Life of Severely Ill ME/CFS and Other Major Diseases. (a) The SF-36 results showed that SIPS patients had significantly lower scores. In particular, scores on Physical functioning (PF), Role limitations due to physical health (RP), General health (GH), Vitality/Energy/Fatigue (VT), and Social functioning (SF) were extremely low, while Role Limitations due to emotional problems (RE) and Mental health/Emotional well-being (MH) were less impacted. (b) Among other major medical conditions, the quality-of-life scores of the SIPS patients were most positively correlated with Congestive Heart Failure (r=0.63) and most negatively correlated with Clinical depression (r=-0.33).

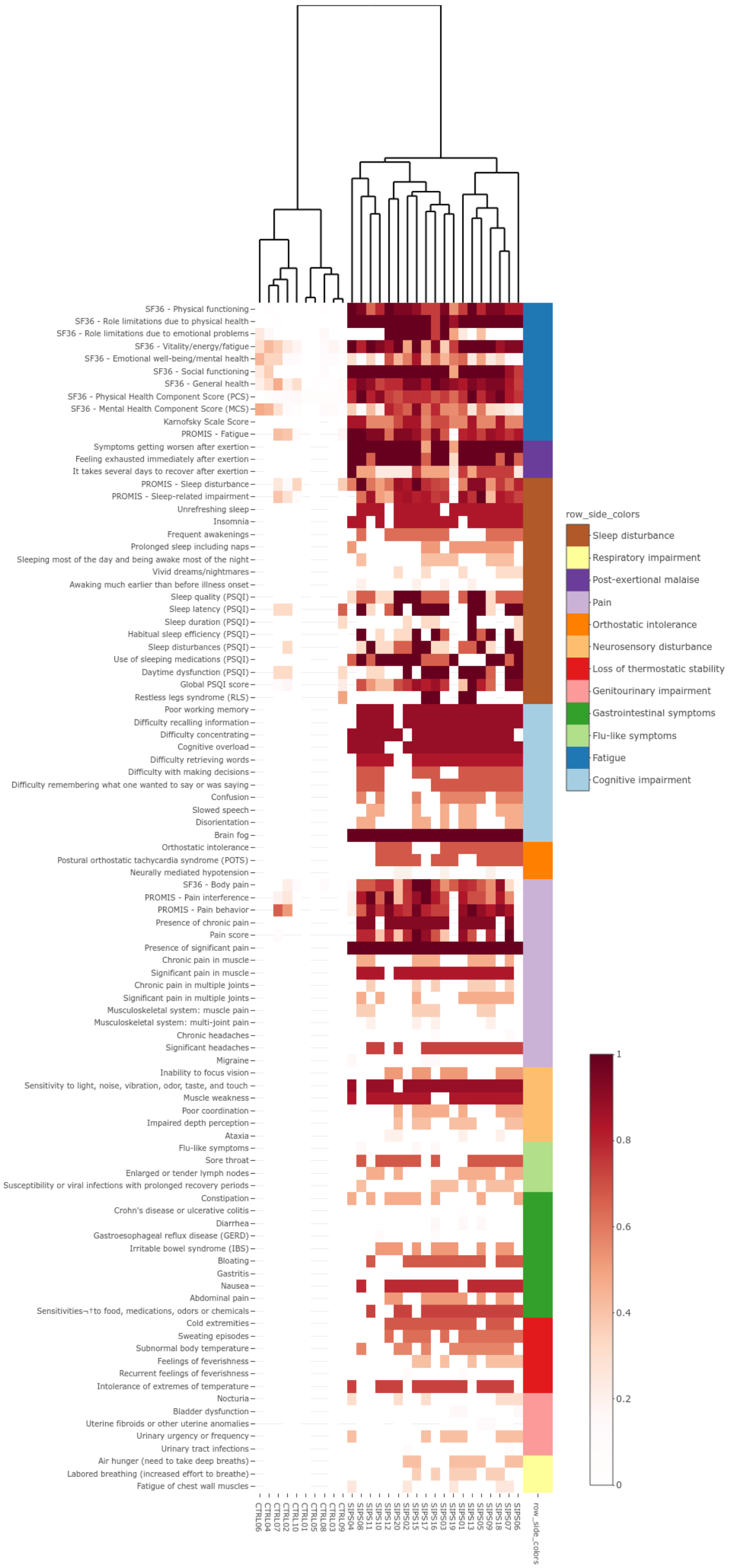

SF36 - Physical functioning
SF36 - Role limitations due to physical health
SF36 - Role limitations due to emotional problems
SF36 - Vitality/energy/fatigue
SF36 - Emotional well-being/mental health
SF36 - Social functioning
SF36 - General health
SF36 - Physical Health Component Score (PCS)
SF36 - Mental Health Component Score (MCS)
Karnofsky Scale Score
PROMIS - Fatigue
Symptoms getting worsen after exertion
Feeling exhausted immediately after exertion
It takes several days to recover after exertion
PROMIS - Sleep disturbance
PROMIS - Sleep-related impairment
Unrefreshing sleep
Insomnia
Frequent awakenings
Prolonged sleep including naps
Sleeping most of the day and being awake most of the night
Vivid dreams/nightmares
Awaking much earlier than before illness onset
Sleep quality (PSQI)
Sleep latency (PSQI)
Sleep duration (PSQI)
Habitual sleep efficiency (PSQI)
Sleep disturbances (PSQI)
Use of sleeping medications (PSQI)
Daytime dysfunction (PSQI)
Global PSQI score
Restless legs syndrome (RLS)
Poor working memory
Difficulty recalling information
Difficulty concentrating
Cognitive overload
Difficulty retrieving words
Difficulty with making decisions
Difficulty remembering what one wanted to say or was saying
Confusion
Slowed speech
Disorientation
Brain fog
Orthostatic intolerance
Postural orthostatic tachycardia syndrome (POTS)
Neurally mediated hypotension
SF36 - Body pain
PROMIS - Pain interference
PROMIS - Pain behavior
Presence of chronic pain
Pain score
Presence of significant pain
Chronic pain in muscle
Significant pain in muscle
Chronic pain in multiple joints
Significant pain in multiple joints
Musculoskeletal system: muscle pain
Musculoskeletal system: multi-joint pain
Chronic headaches
Significant headaches
Migraine
Inability to focus vision
Sensitivity to light, noise, vibration, odor, taste, and touch
Muscle weakness
Poor coordination
Impaired depth perception
Ataxia
Flu-like symptoms
Sore throat
Enlarged or tender lymph nodes
Susceptibility or viral infections with prolonged recovery periods
Constipation
Crohn's disease or ulcerative colitis
Diarrhea
Gastroesophageal reflux disease (GERD)
Irritable bowel syndrome (IBS)
Bloating
Gastritis
Nausea
Abdominal pain
Sensitivities¬†to food, medications, odors or chemicals
Cold extremities
Sweating episodes
Subnormal body temperature
Feelings of feverishness
Recurrent feelings of feverishness
Intolerance of extremes of temperature
Nocturia
Bladder dysfunction
Uterine fibroids or other uterine anomalies
Urinary urgency or frequency
Urinary tract infections
Air hunger (need to take deep breaths)
Labored breathing (increased effort to breathe)
Fatigue of chest wall muscles
CTRL06
CTRL04
CTRL07
CTRL02
CTRL10
CTRL01
CTRL05
CTRL08
CTRL03
CTRL09
SIPS04
SIPS08
SIPS11
SIPS10
SIPS12
SIPS20
SIPS02
SIPS15
SIPS17
SIPS16
SIPS03
SIPS19
SIPS01
SIPS13
SIPS05
SIPS09
SIPS18
SIPS07
SIPS06
row_side_colors
row_side_colors
Sleep disturbance
Respiratory impairment
Post-exertional malaise
Pain
Orthostatic intolerance
Neurosensory disturbance
Loss of thermostatic stability
Genitourinary impairment
Gastrointestinal symptoms
Flu-like symptoms
Fatigue
Cognitive impairment
1
0.8
0.6
0.4
0.2
0

(a)

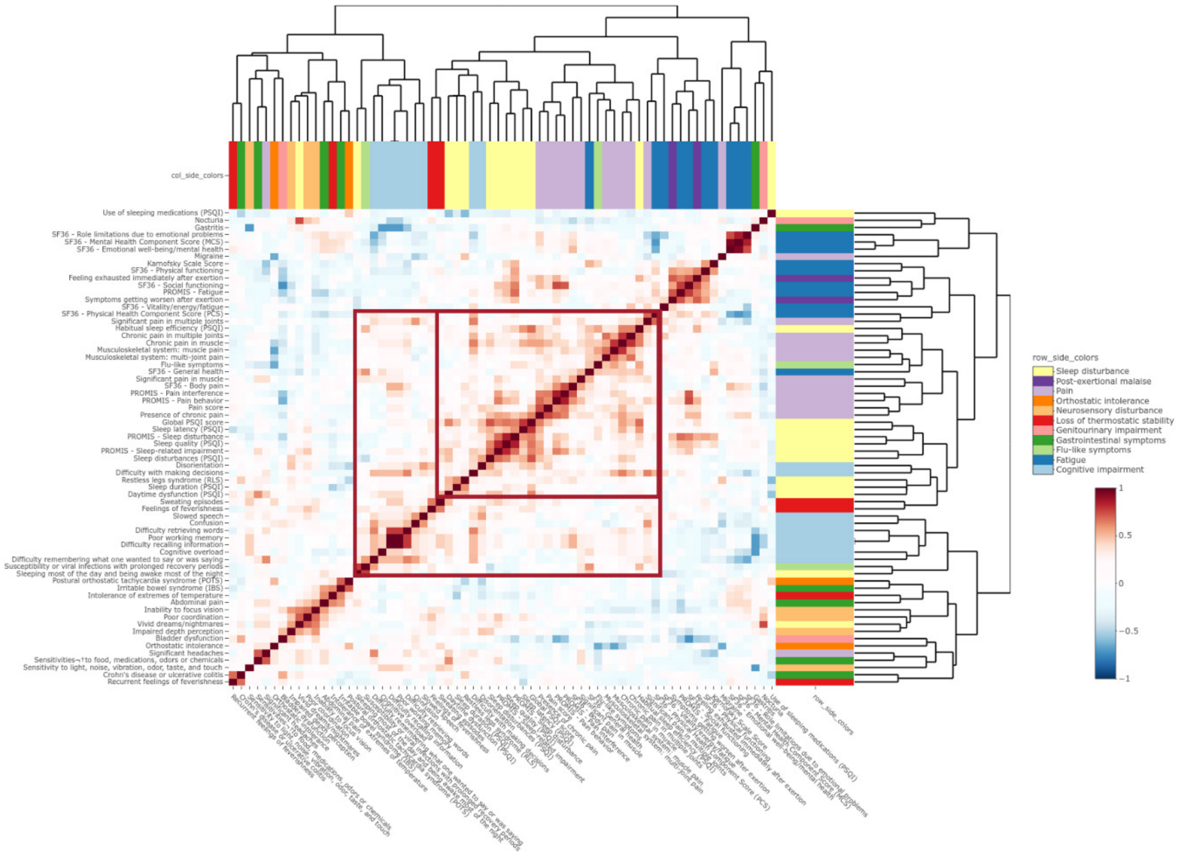

(b)

Figure S2 Similarities and Differences in Clinical Symptoms across 20 SIPS Patients. (a) Shown are results from the questionnaires on whether a patient or a control had a particular condition or the degree of the condition. (b) Hierarchical clustering of these conditions between the patients. Symptoms related to sleep disturbance and symptoms related to pain apparently clustered together.
